# Applications of Indocyanine Green in Breast Cancer for Sentinel Lymph Node Mapping: Protocol for a Scoping Review

**DOI:** 10.2196/66213

**Published:** 2025-01-06

**Authors:** Feryal Kurdi, Yahya Kurdi, Igor Vladimirovich Reshetov

**Affiliations:** 1 Department of Oncology, Radiotherapy and Plastic and Reconstructive Surgery, Sechenov University, Bolshaya Pirogovskaya, 6c1, Moscow, 119021, Russian Federation, 7 9013488810

**Keywords:** indocyanine green, ICG, sentinel lymph node, breast cancer, fluorescence, axillary lymph node mapping, NIR, surgical planning, near-infrared

## Abstract

**Introduction:**

Breast cancer is the leading cause of morbidity and mortality worldwide. Accurate sentinel lymph node (SLN) mapping is crucial for staging and treatment planning in early-stage breast cancer. Indocyanine green (ICG) has emerged as a promising agent for fluorescence imaging in SLN mapping. However, comprehensive assessment of its clinical utility, including accuracy and adverse effects, remains limited. This scoping review aims to consolidate evidence on the use of ICG in breast cancer SLN mapping.

**Objective:**

The objective of this scoping review is to evaluate the current literature on the use of ICG in SLN mapping for patients with breast cancer. This review aims to assess the accuracy, efficacy, and safety of ICG in this context and to identify gaps in the existing research. The outcomes will contribute to the development of further research as part of a PhD project.

**Methods:**

Five electronic databases will be searched (PubMed, Embase, MEDLINE, Web of Science, and Scopus) using search strategies developed in consultation with an academic supervisor. The search strategy is set to human studies published in English within the last 11 years. All retrieved citations will be imported to Zotero and then uploaded to Covidence for the screening of titles, abstracts, and full text according to prespecified inclusion criteria. Patients with early-stage breast cancer (T1 and T2), selected T3 cases where the SLN biopsy is accurate, and those with clinically node-negative breast cancer will be included. The intervention criterion includes studies using ICG for SLN mapping and studies on the assessment of fluorescence imaging cameras. Citations meeting the inclusion criteria for full-text review will have their data extracted by 2 independent reviewers, with disagreements resolved by discussion. A data extraction tool will be developed to capture full details about the participants, concept, and context, and findings relevant to the scoping review will be summarized.

**Results:**

The preliminary search began in December 2023. As of September 2024, papers have been screened and data are currently being extracted. Out of the 2130 references initially imported, 126 studies met the inclusion criteria after screening. The scoping review is expected to be published in January 2025.

**Conclusions:**

Although ICG technology has been used for SLN mapping in patients with breast cancer, initial searches in 2022 revealed limited data on this technique’s feasibility, safety, and effectiveness. At that time, preliminary search of Scopus, MEDLINE, Embase, and PubMed identified no current or forthcoming systematic reviews or scoping reviews on the topic. However, recent searches indicate a substantial increase in research and reviews, reflecting a growing interest and evidence in this area.

## Introduction

Sentinel lymph node (SLN) biopsy plays a crucial role in staging and prognosis in breast cancer management. The SLN is the initial lymph node to which breast cancer cells are likely to metastasize, and the presence of cancer cells in the SLN indicates a higher likelihood of further metastasis to other lymph nodes and distant organs [1].

SLN biopsy involves injecting a tracer substance into the breast, which then migrates to the SLN. The SLN is then identified, excised, and examined for cancer cells. If the SLN is free of cancer cells, it suggests that the cancer has not spread to other lymph nodes, eliminating the need for additional lymph node dissection. Conversely, if the SLN contains metastases, further dissection is typically required [2].

Over the past 2 decades, SLN biopsy using blue dye and radiotracers has been established as the diagnostic standard of care for patients with early-stage breast cancer who have clinically negative lymph nodes [3,4].

However, these methods come with certain drawbacks, including the potential for allergic reactions to the blue dye and the necessity of nuclear medicine facilities for radiotracer injection and detection. In a cohort undergoing blue dye and radiotracer injection procedures, a small number of adverse reactions, such as skin tattooing and anaphylaxis, were reported [5].

In recent years, near-infrared (NIR) fluorescence imaging using indocyanine green (ICG) has emerged as an alternative approach for SLN mapping in patients with breast cancer. ICG, a fluorescent dye, is injected into the breast, which then migrates to the SLNs. A NIR camera detects the fluorescence emitted by ICG, enabling the surgeon to identify and excise the SLNs [6,7].

This technology offers several advantages over traditional methods, including enhanced visualization of SLNs, a lower risk of allergic reactions, and the elimination of the need for nuclear medicine facilities. Furthermore, ICG has an excellent safety profile [8-11].

The importance of this topic stems from the potential of ICG technology to enhance the accuracy and safety of SLN mapping in patients with breast cancer. Precise identification and removal of the SLN are crucial for accurate staging and prognosis. Inaccurate SLN identification can lead to unnecessary lymph node dissection, resulting in complications such as lymphedema and impaired arm function. Sampling a larger number of SLNs may increase the risk of upper limb lymphedema, sensory deficits, and reduced shoulder function.

Landmark trials have shown a significant difference in morbidity rates when comparing SLN biopsy to axillary dissection, with rates of 25% and 70%, respectively [3,12]. Recent studies have reported excising, on average, 2 nodes per patient, likely due to advancements in NIR technology and ICG fluorescence protocols [13-17]. Nevertheless, further research is essential to assess the long-term outcomes and cost-effectiveness of ICG technology compared to traditional methods.

## Methods

### Overview

The proposed scoping review will be guided by the JBI methodology for scoping reviews [18]. The search strategy aims to locate both published and unpublished articles. An initial limited search of PubMed, Embase, MEDLINE, Web of Science, and Scopus was undertaken to identify relevant articles on the use of ICG for SLN mapping in breast cancer. In consultation with an academic supervisor, the keywords in the titles and abstracts of relevant articles, as well as the index terms used to describe these articles, were used to develop a comprehensive search strategy for PubMed, Embase, MEDLINE, Web of Science, and Scopus (see Multimedia Appendix 1). This strategy, including all identified keywords and index terms, will be adapted for each included database. The articles sourced from all included sources of evidence will be exported into Zotero (Corporation for Digital Scholarship).

Only articles published in English will be included due to the language proficiency of the reviewers. Articles published since January 1, 2014, will be included to ensure relevance, aligning with the project’s consideration of recent data and the ongoing advancements in SLN mapping techniques using ICG.

### JBI Methodology for Scoping Reviews

The outcomes of the scoping review will inform and frame three subsequent pieces of work planned as part of a PhD project:

1. Prospective cohort study on the long-term outcomes of ICG in SLN mapping
2. Systematic review and meta-analysis of ICG for SLN mapping in breast cancer
3. Development of standardized clinical guidelines and protocols for the use of ICG in SLN mapping in patients with breast cancer

The Participants-Concept-Context framework for this scoping review defines (1) the participants as patients with early-stage breast cancer, (2) the concept as the use of ICG for SLN mapping in patients with breast cancer, and (3) the context as SLN mapping that is performed as part of breast cancer staging and treatment planning.

### Review Questions

The review questions are as follows:

1. What do we know about the evaluation and integration of emergent evidence on the use of ICG for SLN mapping in patients with breast cancer into clinical practice and decision-making?
2. To what extent is emergent evidence on the feasibility, safety, and effectiveness of ICG for SLN mapping integrated into clinical guidelines and decision-making processes?
3. How is emergent evidence on the use of ICG for SLN mapping evaluated and incorporated into clinical guidelines and decision-making processes?

For the purposes of this scoping review, emergent evidence refers to new research findings on ICG for SLN mapping that have emerged after market launch and have not yet been fully integrated into clinical guidelines and practice.

### Eligibility Criteria

The eligibility criteria are as follows. Participants will include patients with early-stage breast cancer (T1 and T2) and selected T3 cases where SLN biopsy has been shown to be accurate. Concept will include the use of ICG for SLN mapping in patients with breast cancer, as well as the assessment of imaging techniques and devices used in conjunction with ICG for SLN mapping. Context will include clinical settings where SLN mapping is performed as part of breast cancer staging and treatment planning.

This scoping review will consider both experimental and quasi-experimental study designs, including controlled before-and-after studies and controlled interrupted time-series studies. In addition, analytical observational studies including prospective and retrospective cohort studies, case-control studies, and analytical cross-sectional studies will be considered for inclusion. This review will also consider descriptive observational study designs such as descriptive cross-sectional studies for inclusion. Qualitative studies that focus on qualitative data will be considered for inclusion.

Following the search, all identified articles will be exported into Zotero. Then, the remaining articles will be uploaded into Covidence (Veritas Health Innovations Ltd). Titles and abstracts will then be screened by the lead author against the inclusion criteria for the scoping review. Potentially relevant articles will be retrieved in full and included in Covidence. The full text of these articles will be assessed in detail against the inclusion criteria by 2 independent reviewers. Reasons for the exclusion of sources of evidence at the full-text stage that do not meet the inclusion criteria will be recorded and reported in the scoping review. Any disagreements that arise between the reviewers at each stage of the selection process will be resolved through discussion or with an additional reviewer. The results of the search and the study inclusion process will be reported in full in the final scoping review and presented in a PRISMA-ScR (Preferred Reporting Items for Systematic Reviews and Meta-Analyses Extension for Scoping Reviews) checklist, as extracted from Covidence (see Multimedia Appendix 2) [19].

Data will be extracted from all articles included in the scoping review by 2 independent reviewers, using a data extraction tool developed by the lead reviewer and piloted with about 15 articles to refine and improve it. The data extracted will include specific details about the participants, concept, context, study methods, and key findings relevant to the scoping review questions and will be imported into either Covidence or Microsoft Excel.

A draft extraction form is provided (see Multimedia Appendix 3). The draft data extraction tool will be modified and revised as necessary during the process of extracting data from each included article. Modifications will be detailed in the scoping review. Any disagreements that arise between the reviewers will be resolved through discussion or with an additional reviewer. If appropriate, authors of articles will be contacted to request missing or additional data, where required.

The evidence presented will directly respond to the scoping review’s objective and questions. The data will be presented graphically or in diagrammatic or tabular form. A narrative summary will accompany the tabulated and/or charted results and will describe how the results relate to the scoping review’s objective and questions.

## Results

The preliminary search began in December 2023. As of September 2024, papers have been screened and data are currently being extracted. Out of the 2130 references initially imported, 126 studies met the inclusion criteria after screening (see Multimedia Appendix 4). The scoping review is anticipated to be published in January 2025.

## Discussion

The significance of SLN mapping using ICG technology in breast cancer lies in its potential to enhance accuracy and safety, reduce complications, and improve patient outcomes [20]. Although ICG technology has been used for SLN mapping in patients with breast cancer, initial searches in 2022 revealed limited data on the feasibility, safety, and effectiveness of this technique. At that time, a preliminary search of Scopus, MEDLINE, Embase, and PubMed identified no current or forthcoming systematic reviews or scoping reviews on the topic. However, recent searches indicate a substantial increase in research and reviews, reflecting a growing interest and evidence in this area. Further studies are necessary to assess the long-term efficacy and cost-effectiveness of this technique and to identify the patient populations most likely to benefit.

The objective of this scoping review is to assess the extent of the literature on SLN mapping using ICG technology around the evaluation and integration of emergent evidence for benefits and harms; explore its feasibility, safety, and effectiveness in a larger cohort of patients with breast cancer; and provide guidance for clinical decision-making.

This scoping review could also identify specific patient populations, such as those with higher BMIs, who may benefit most from ICG technology. Additionally, patients who have undergone neoadjuvant therapy could be particularly advantageous candidates.

Factors such as the type of NIR cameras used, the learning curve for surgeons to become proficient with ICG for SLN detection, the availability of ICG and radioisotopes, the presence of nuclear medicine facilities, regional variations in ICG usage, and cost comparisons with the gold standard are also critical considerations in the broader adoption of this technology.

Limitations of this study include a lack of quantitative synthesis (ie, meta-analysis) of the results, which may limit the ability to draw strong conclusions. This scoping review serves as a foundational step toward a more comprehensive systematic review and meta-analysis guiding the clinical decision-making and the integration of ICG into standardized guidelines for SLN mapping in patients with breast cancer.

## Supplementary material

10.2196/66213 Multimedia Appendix 1 Search strategy.

10.2196/66213 Multimedia Appendix 2 PRISMA-ScR (Preferred Reporting Items for Systematic Reviews and Meta-Analyses Extension for Scoping Reviews) checklist.

10.2196/66213 Multimedia Appendix 3 Data extraction instrument.

10.2196/66213 Multimedia Appendix 4 PRISMA (Preferred Reporting Items for Systematic Reviews and Meta-Analyses) extraction flowchart.

## Abbreviations

ICG: indocyanine green
NIR: near-infrared
PRISMA-ScR: Preferred Reporting Items for Systematic Reviews and Meta-Analyses Extension for Scoping Reviews
SLN: sentinel lymph node
