# Supplementary material for: Applications of Indocyanine Green in Breast Cancer for Sentinel Lymph Node Mapping: Protocol for a Scoping Review
Source: JMIRx Med. 2025 Jan 6;6:e66213. doi: 10.2196/66213 (PMC11728196; doi:10.2196/66213)
Supplement: Multimedia Appendix 1 [file xmed-v6-e66213-s001.docx]

### **Appendix 1: Search strategy**

Search strategies will be developed in consultation with an academic supervisor for PubMed, EMBASE, MEDLINE, Web of Science, and SCOPUS. All search strategies will be available on request.

Database: Embase

('indocyanine green'/exp OR 'indocyanine green' OR

(('indocyanine'/exp OR indocyanine) AND ('green'/exp OR green))

OR 'icg'/exp OR icg OR 'fluorescence imaging'/exp OR 'fluorescence imaging'

OR (('fluorescence'/exp OR fluorescence) AND ('imaging'/exp OR imaging)))

AND

('sentinel lymph node mapping'/exp OR 'sentinel lymph node mapping' OR

(('sentinel'/exp OR sentinel) AND ('lymph'/exp OR lymph) AND node

AND ('mapping'/exp OR mapping)) OR 'sentinel lymph node biopsy'/exp

OR 'sentinel lymph node biopsy' OR (('sentinel'/exp OR sentinel)

AND ('lymph'/exp OR lymph) AND node AND ('biopsy'/exp OR biopsy))

OR 'sentinel node biopsy'/exp OR 'sentinel node biopsy'

OR (('sentinel'/exp OR sentinel) AND node AND ('biopsy'/exp OR biopsy))

OR 'sentinel node' OR (('sentinel'/exp OR sentinel) AND node))

AND

('breast cancer'/exp OR 'breast cancer' OR

(('breast'/exp OR breast) AND ('cancer'/exp OR cancer))

OR 'breast neoplasm'/exp OR 'breast neoplasm' OR

(('breast'/exp OR breast) AND ('neoplasm'/exp OR neoplasm))

OR 'breast carcinoma'/exp OR 'breast carcinoma' OR

(('breast'/exp OR breast) AND ('carcinoma'/exp OR carcinoma)))

NOT

('lung cancer'/exp OR 'lung cancer' OR

(('lung'/exp OR lung) AND ('cancer'/exp OR cancer))

OR 'cervical cancer'/exp OR 'cervical cancer' OR

(cervical AND ('cancer'/exp OR cancer))

OR 'endometrial cancer'/exp OR 'endometrial cancer'

OR (endometrial AND ('cancer'/exp OR cancer)))

AND

[english]/lim AND [humans]/lim

Limiters: Human; English

Publication Date: Last 11 years
