# Supplementary material for: Applications of Indocyanine Green in Breast Cancer for Sentinel Lymph Node Mapping: Protocol for a Scoping Review
Source: JMIRx Med. 2025 Jan 6;6:e66213. doi: 10.2196/66213 (PMC11728196; doi:10.2196/66213)
Supplement: Multimedia Appendix 3 [file xmed-v6-e66213-s003.docx]

### **Appendix 3: Data extraction instrument**

The general characteristics of all included articles will be recorded and presented in a descriptive table or other format that may include but is not limited to the following:

- Author(s)
- Year of publication
- Source/Origin/country of origin
- Aims/purpose
- Study population and sample size
- Methodology
- Intervention type and comparator
- Concept
- Duration of the intervention
- How outcomes are measured
- Key findings that relate to the RQ

This descriptive table will be separated into two broad categories of interest: ICG and other tracers. It will be piloted with about 15 articles to ensure all relevant results are included and modified if needed.
