# Supplementary material for: Applications of Indocyanine Green in Breast Cancer for Sentinel Lymph Node Mapping: Protocol for a Scoping Review
Source: JMIRx Med. 2025 Jan 6;6:e66213. doi: 10.2196/66213 (PMC11728196; doi:10.2196/66213)
Supplement: Multimedia Appendix 4 [file xmed-v6-e66213-s004.docx]

Studies from databases/registers **(n = 2130)**

Scopus (n = 832)

Embase (n = 524)

Web of Science (n = 366)

PubMed (n = 260)

MEDLINE (n = 148)

References from other sources **(n = )**

Citation searching (n = )

Grey literature (n = )

**Identification**

Included studies ongoing **(n = 0)**

Studies awaiting classification **(n = 0)**

Studies included in review **(n = 126)**

Studies excluded **(n = 1179)**

Studies not retrieved **(n = 0)**

Studies assessed for eligibility **(n = 272)**

Studies sought for retrieval **(n = 272)**

Studies screened **(n = 1451)**

Studies excluded **(n = 146)**

Wrong setting (n = 14)

Wrong Language (n = 8)

Wrong outcomes (n = 1)

Wrong comparator (n = 1)

Wrong intervention (n = 16)

Wrong study design (n = 4)

Wrong patient population (n = 1)

Wrong Study Characteristic (n = 101)

References removed **(n = 679)**

Duplicates identified manually (n = 1)

Duplicates identified by Covidence (n = 678)

Marked as ineligible by automation tools (n = 0)

Other reasons (n = )

**Screening**

**Included**
